# Dietary fatty acids sex-specifically modulate guinea pig postnatal development via cortisol concentrations

**DOI:** 10.1038/s41598-017-18978-4

**Published:** 2018-01-11

**Authors:** Matthias Nemeth, Eva Millesi, Daniela Schuster, Ruth Quint, Karl-Heinz Wagner, Bernard Wallner

**Affiliations:** 1 Department of Behavioural Biology, Faculty of Life Sciences, University of Vienna, Althanstrasse 14, 1090 Vienna, Austria; 2 Department of Nutritional Sciences, Faculty of Life Sciences, University of Vienna, Althanstrasse 14, 1090 Vienna, Austria; 3 Department of Anthropology, Faculty of Life Sciences, University of Vienna, Althanstrasse 14, 1090 Vienna, Austria

## Abstract

Early ontogenetic periods and postnatal maturation in organisms are sex-specifically sensitive to hypothalamic-pituitary-adrenal (HPA)-axis activities, related glucocorticoid secretions, and their effects on energy balance and homeostasis. Dietary polyunsaturated (PUFAs) and saturated (SFAs) fatty acids potentially play a major role in this context because PUFAs positively affect HPA-axis functions and a shift towards SFAs may impair body homeostasis. Here we show that dietary PUFAs positively affect postnatal body mass gain and diminish negative glucocorticoid-effects on structural growth rates in male guinea pigs. In contrast, SFAs increased glucocorticoid concentrations, which positively affected testes size and testosterone concentrations in males, but limited their body mass gain and first year survival rate. No distinct diet-related effects were detectable on female growth rates. These results highlight the importance of PUFAs in balancing body homeostasis during male’s juvenile development, which clearly derived from a sex-specific energetic advantage of dietary PUFA intakes compared to SFAs.

## Introduction

Early developmental processes in humans and animal models are highly dependent on environmental and nutritional conditions, which determine the progress of structural and functional changes in the body and enable a balanced energy homeostasis throughout lifetime^1–3^. This is primarily achieved via hypothalamic-pituitary-adrenal (HPA)-axis functions and the modulation of metabolic processes, immune responses, and energy balance by glucocorticoids such as cortisol^4,5^. In this context, adequate dietary fat intakes and ratios of polyunsaturated (PUFAs) to saturated (SFAs) fatty acids are suggested to be highly important. Long-chain metabolites of the dietary essential omega-3 (n-3) and omega-6 (n-6) PUFAs alpha-linolenic acid (18:3 n-3) and linoleic acid (18:2 n-6), including docosahexaenoic acid (22:6 n-3), eicosapentaenoic acid (20:5 n-3), and arachidonic acid (20:4 n-6), are key components of neuronal cell membranes. They can positively affect neuronal development and neurotransmission^6^, which apparently also applies to the hypothalamus and the HPA-axis^7,8^.

Especially n-3 PUFAs have repeatedly been shown to be highly important regarding adequate HPA-axis functions. Adequate and elevated dietary intakes diminish glucocorticoid secretion rates and lower vulnerabilities to stress-induced metabolic and behavioural impairments in different rodent models as well as in humans^9–13^. The general bioavailability of n-3 PUFAs in different tissues and the PUFA:SFA ratio play a crucial role in this context because dietary n-3 PUFA deficiencies or elevated intakes of SFAs can elicit excessive glucocorticoid secretion rates and impair physiological and metabolic responses to stress^14–17^. The illustrated opposite effects of PUFAs and SFAs on HPA-axis functions and glucocorticoid-related influences on metabolic processes could also be related to different beta-oxidation rates^18^, effects on cholesterol metabolism^19^, and steroidogenesis in general^20^. PUFAs and SFAs may therefore also differently contribute to whole body homeostasis and energy balance via indirect effects on glucocorticoid secretion rates, instead of primarily targeting the neuronal control of the HPA-axis. Importantly, HPA-axis dysfunctions caused by adverse environmental or nutritional conditions during early ontogenetic periods can strongly impair developmental processes by evoking brain- and metabolic-related diseases up until adulthood^1,2,14,21^.

Sensitive ontogenetic periods such as the early postnatal development and maturation are characterized by pronounced changes in glucocorticoid and other steroid hormone concentrations^22,23^. Studies on domestic guinea pigs revealed that early ontogenetic influences on HPA-axis functions via prenatal stress exposure or altered nutritional conditions can exert sex-specific effects on growth rates linked to the structural and functional development of the HPA-axis and cortisol concentrations^22,24–27^. These responses point to sex-specific energetic requirements during early ontogenetic periods, which could be modulated via sexually different cortisol secretion rates and effects on the energy metabolism. Recent findings suggest that sex-specific saliva cortisol secretion rates and cortisol responses resulted in sex-specific effects of dietary fatty acids on cognitive and behavioural performances in adult guinea pigs^17,28^. Dietary fatty acid intakes may therefore significantly contribute to sexually different HPA-axis functions by altering circulating cortisol levels and the energy balance in general and perhaps also by compensating sex-specific energetic needs. Consequently, dietary fatty acid intakes could sex-specifically influence postnatal developmental processes and adult outcomes. We test this hypothesis by determining and comparing the effects of dietary PUFAs and SFAs on the structural growth in male and female guinea pigs during postnatal maturation and linking this to cortisol levels as indicators of whole body homeostasis.

This study was performed in male and female guinea pigs belonging to a F_1_ generation of animals maintained on a control, high-PUFA, or high-SFA diet throughout. We initially monitored saliva cortisol concentrations as indicators of homeostasis, as well as body mass and head length as measurements of the structural growth from weaning until 120 days of age (the onset of adulthood) and compared their changes in time between sexes and dietary groups. Structural measurements were analysed in relation to cortisol concentrations in order to determine potential cortisol-related effects on the structural growth. Additional measurements of testes width and plasma testosterone concentrations in males served as indicators of testes development and were further integrated in male-specific analyses of the body mass gain with age. Intrauterine influences of PUFAs and SFAs have not been implicated in effects on the natal body mass in guinea pigs so far, but an altered reproductive performance and output have been documented^29^. We therefore additionally controlled for litter size effects and corrected all analyses for the individual’s relatedness. Plasma fatty acid patterns were analysed at 120 days of age to determine the integration of these molecules in metabolic processes during the maturation period. Finally, we monitored the first-year survival rate of the experimental animals as potential long-term indicators of diet-induced effects on homeostasis and health until adulthood. An indication of any sex-specific effects of dietary PUFAs and SFAs on growth rates or on HPA-axis functions, as well as glucocorticoid-related effects on structural growth, would argue for sexually different requirements for these nutrients in order to cope with the energetically demanding postnatal developmental and sexual maturation period and balancing homeostasis throughout lifetime.

## Results

### Saliva cortisol concentrations

**Figure 1:**
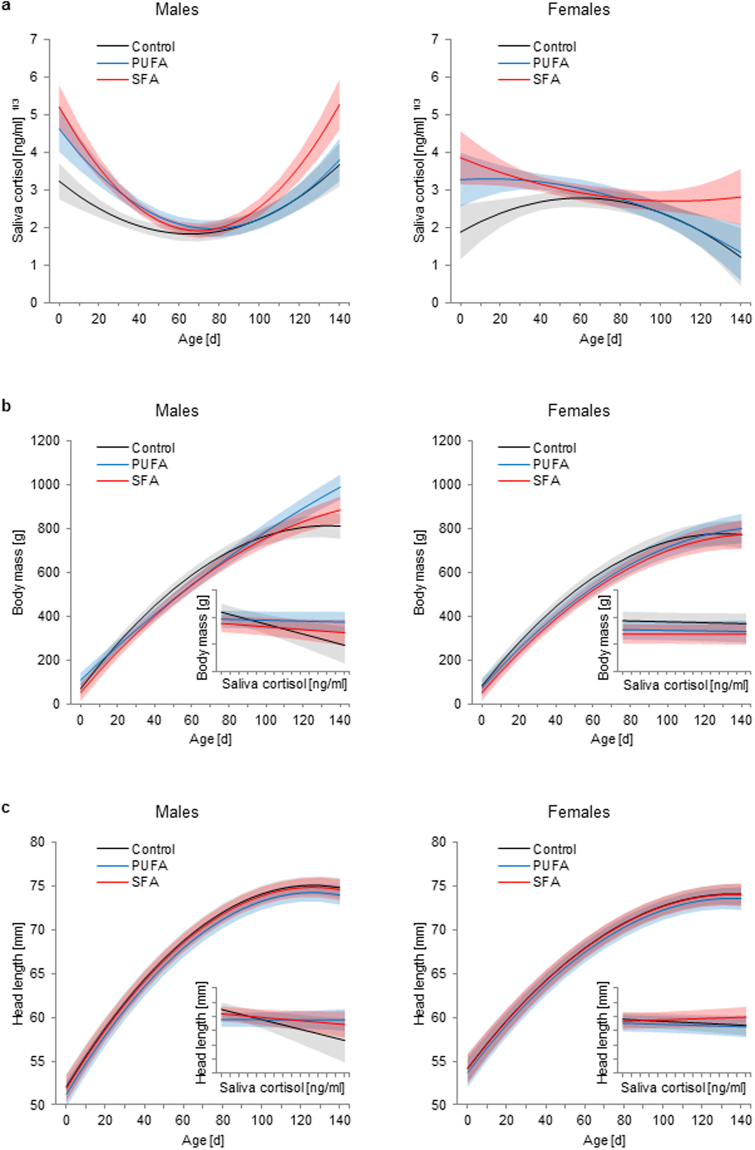
Juvenile development in male and female guinea pigs maintained on a control, high-PUFA, or high-SFA diet. (**a**) Effects of age on saliva cortisol concentrations (number of observations: 561). (**b**) Effects of age and saliva cortisol concentrations (insert graph; saliva cortisol: 0–140 ng/ml, body mass: 400–700 g) on body mass (number of observations: 561). (**c**) Effects of age and saliva cortisol concentrations (insert graph; saliva cortisol: 0–140 ng/ml, head length: 62–74 mm) on head length (number of observations: 475). (**a**–**c**) Filled areas represent 95% confidence intervals. Sample sizes: control: 14♂, 10♀; PUFA: 14♂, 9♀; SFA: 13♂, 9♀. Mean number of repeated measurements: 8.13 (cortisol and body mass), 6.88 (head length). Statistics and effect sizes are outlined in Table 1.

Saliva cortisol concentrations were significantly affected by diet and sex in relation to the animals’ age (diet:age: *F*_4,484_ = 7.531, *p* < 0.001; sex:age: *F*_2,484_ = 36.231, *p* < 0.001). Males in general showed a U-shaped pattern in saliva cortisol secretion rates with age and a steep increase in the second half of the observation period, while females showed decreasing levels (Fig. 1a). The interaction effect of diet and age, however, revealed that saliva cortisol concentrations in SFA animals increased most strongly with age. This is indicated by the highest positively pronounced quadratic effect of age, which represents the deviation from linearity and therefore the nonlinear changes with age (Table 1). This resulted in the highest cortisol levels in SFA animals, but especially in SFA males at an age of 120 days (the end of the observation period); cortisol levels were similarly lower in control and PUFA animals (Fig. 1a, Table 2).

**Table 1:** Model coefficients for male and female guinea pigs maintained on a control, high-PUFA, or high-SFA diet.

Model	Sex	Diet			Contrasts (p-values)	
		Control	PUFA	SFA	C vs. P	C vs. S
**Cortisol** ^**1/3**^						
Age1	male	−0.0430 ± 0.0081***^#^	−0.0696 ± 0.0119***^#^	−0.0948 ± 0.0119***^#^	0.025	<0.001
	female	0.0301 ± 0.0119*	0.0036 ± 0.0119	−0.0216 ± 0.0119	0.025	<0.001
Age2	male	0.00033 ± 0.00005***^#^	0.00046 ± 0.00008***^#^	0.00068 ± 0.00008***^#^	0.122	<0.001
	female	−0.00025 ± 0.00008**	−0.00012 ± 0.00008	0.00010 ± 0.00008	0.122	<0.001
**Body mass**						
Age1	male	11.182 ± 0.389***	7.993 ± 0.519***^#^	9.824 ± 0.649***	<0.001	0.036
	female	10.719 ± 0.498***	9.477 ± 0.704***	9.784 ± 0.784***	0.030	0.075
Age2	male	−0.0419 ± 0.0023***	−0.0122 ± 0.0029***^#^	−0.0277 ± 0.0041***	<0.001	<0.001
	female	−0.0414 ± 0.0030 ***	−0.0307 ± 0.0043***	−0.0331 ± 0.0050***	<0.001	0.002
Cortisol	male	−0.8768 ± 0.2410***^#^	−0.0797 ± 0.2590	−0.2479 ± 0.2881	0.002	0.029
	female	−0.0754 ± 0.2516	−0.0469 ± 0.2835	−0.0038 ± 0.3069	0.805	0.499
**Head length**						
Age1	male	0.3640 ± 0.0123***^#^	0.3640 ± 0.0123***^#^	0.3640 ± 0.0123***^#^	n.a.	n.a.
	female	0.2940 ± 0.0173***	0.2940 ± 0.0173 ***	0.2940 ± 0.0173***	n.a.	n.a.
Age2	male	−0.0014 ± 0.0001 ***^#^	−0.0014 ± 0.0001***^#^	−0.0014 ± 0.0001***^#^	n.a.	n.a.
	female	−0.0011 ± 0.0001***	−0.0011 ± 0.0001***	−0.0011 ± 0.0001***	n.a.	n.a.
Cortisol	male	−0.0310 ± 0.0117**^#^	−0.0003 ± 0.0125	−0.0107 ± 0.0132	0.014	0.124
	female	−0.0062 ± 0.0124	−0.0037 ± 0.0138	0.0041 ± 0.0144	0.664	0.074
**Testes width**						
Age1	male	0.3614 ± 0.0198***	0.3614 ± 0.0198***	0.3614 ± 0.0198***	n.a.	n.a.
Age2	male	−0.00144 ± 0.00011***	−0.00144 ± 0.00011***	−0.00144 ± 0.00011***	n.a.	n.a.
Cortisol	male	−0.0223 ± 0.0106*	−0.0047 ± 0.0122	0.0122 ± 0.0135	0.147	0.010
**Testosterone** ^**1/2**^						
Age1	male	0.0759 ± 0.0242**	0.0759 ± 0.0242**	0.0759 ± 0.0242**	n.a.	n.a.
	female	−0.0021 ± 0.0267	−0.0021 ± 0.0267	−0.0021 ± 0.0267	n.a.	n.a.
Age2	male	−0.00045 ± 0.00014**	−0.00045 ± 0.00014**	−0.00045 ± 0.00014**	n.a.	n.a.
	female	−0.00001 ± 0.00015	−0.00001 ± 0.00015	−0.00001 ± 0.00015	n.a.	n.a.
**Male body mass 1**						
Age1	male	10.797 ± 1.207***	9.458 ± 1.968***	10.963 ± 2.167***	0.496	0.939
Age2	male	−0.0390 ± 0.0065***	−0.0198 ± 0.0106*	−0.0354 ± 0.0130**	0.072	0.267
Cortisol	male	−1.3283 ± 0.4275**	−0.2576 ± 0.5620	−0.0559 ± 0.5917	0.057	0.032
**Male body mass 2**						
Age1	male	9.972 ± 2.097***	6.904 ± 3.303**	6.947 ± 3.299**	0.358	0.364
Age2	male	−0.0394 ± 0.0109***	−0.0114 ± 0.0165	−0.0135 ± 0.0180	0.097	0.156
Cortisol	male	−0.909 ± 5.803	6.303 ± 8.568	−15.735 ± 7.000***	0.405	0.04
Testosterone	male	−7.736 ± 9.642	−0.761 ± 16.440	31.370 ± 14.618***	0.673	0.01
Testes	male	8.825 ± 5.668	14.316 ± 9.023	14.672 ± 8.433	0.546	0.492
Cortisol: Testes	male	−0.0109 ± 0.1944	−0.2230 ± 0.2811	0.4416 ± 0.2275***	0.455	0.053
Testosterone: Testes	male	0.3428 ± 0.3274	−0.0273 ± 0.5378	−1.0051 ± 0.4942**	0.495	0.009

Age1: linear effect of age.

Age2: quadratic effect of age.

Contrasts represent comparisons of PUFA (P) and SFA (S) groups to the control (C) group.

*p ≤ 0.05, **p ≤ 0.01, ***p ≤ 0.001 for significant effect of the respective diet-sex group.

^#^*p* ≤ 0.05 comparing males and females within a dietary regime.

n.a.: not available because diet had no significant effect and was removed from the respective calculations based on the AIC.

Male body mass 1 & 2 correspond to calculations and effects on male-specific body mass changes based on a reduced dataset.

**Table 2:** Predicted values [+95% confidence limits] for 120 day of age of the measured parameters in male and female guinea pigs maintained on a control, high-PUFA, or high-SFA diet.

Measurement	Sex	Diet		
		Control	PUFA	SFA
Saliva cortisol (ng/ml)^a^	male	22.22 [15.01, 31.44]	22.47 [15.20, 31.75]	47.79 [34.23, 64.51]
	female	6.82 [3.13, 12.65]	6.93 [3.24, 12.72]	19.99 [12.18, 30.59]
Body mass (g)	male	805 [757, 853]	892 [844, 941]	831 [783, 880]
	female	772 [720, 825]	769 [713, 825]	748 [694, 803]
Head length (mm)	male	75.0 [74.0, 76.0]	74.2 [73.2, 75.2]	74.8 [73.7, 75.9]
	female	73.8 [72.7, 74.9]	73.3 [72.2, 74.5]	73.8 [72.6, 74.9]
Testes width (mm)	male	33.02 [32.25, 33.79]	32.55 [31.77, 33.33]	31.99 [31.10, 32.88]
Plasma testosterone (ng/ml)^a^	male	4.85 [2.78, 6.91]	6.38 [4.48, 8.28]	8.32 [6.23, 10.42]
	female	0.34 [0.11, 0.58]	0.47 [0.14, 0.80]	0.23 [0.06, 0.41]
Male body mass model 1 (g)	male	807 [763, 852]	880 [835, 925]	844 [791, 897]
Male body mass model 2 (g)	male	778 [733,823]	854 [810, 898]	848 [791, 904]

Values were extracted from linear mixed models on the respective parameters, corrected for saliva cortisol concentrations and litter size, with age and individual ID within mother as random effects.

^a^Back-transformed values from second and third root transformations.

Male body mass model 1 corresponds to calculations of male-specific body mass based on a reduced dataset.

Male body mass model 2 corresponds to calculations of male-specific body mass based on a reduced dataset, additionally corrected for testes development.

### Body mass

The body mass gain with age was sex-specifically affected by diet (diet:sex:age: *F*_4,474_ = 6.237, *p* < 0.001). Moreover, saliva cortisol concentrations also showed an interaction effect with diet and sex on the body mass (diet:sex:cortisol: *F*_2,474_ = 3.701, *p* = 0.025), and litter size in general negatively affected body mass (*F*_1,40_ = 13.430, *p* < 0.001).

Body mass increased more linearly in PUFA and SFA animals, while control animals showed a stronger increase in the first months (strongest positively pronounced linear effect of age), but reached the plateau phase earlier (strongest negatively pronounced quadratic effect of age) (Fig. 1b, Table 1). Although these age-related differences in body mass occurred in both sexes, PUFA males showed the least pronounced linear and quadratic effects of age (Table 1), resulting in the most linear body mass gain in these animals and the highest body mass at an age of 120 days (Fig. 1b, Table 2). As differences in these age-effects were less pronounced among females (Table 1), they exhibited similar body masses in the end, irrespective of diet (Fig. 1b, Table 2). Body mass gain of PUFA males also differed to that of PUFA females (Table 1). No such sex difference was detected in control and SFA animals, but males generally reached a higher body mass than females (Table 2).

Saliva cortisol concentrations negatively affected the body mass of control males (Fig. 1b insert graph, Table 1). This effect was significantly diminished in PUFA and SFA males, insofar as their body mass remained unaffected by saliva cortisol concentrations (Fig. 1b insert graph, Table 1). Also, all female groups appeared to be unaffected by their cortisol levels, and therefore a sex-difference regarding this effect was detected in control animals, while no differences occurred among females or between sexes in the fatty acid groups (Fig. 1b insert graph, Table 1).

### Head length

The increase in head length with age differed only between the sexes (sex:age: *F*_2,396_ = 10.401, *p* < 0.001), whereas cortisol interacted with diet and sex again (diet:sex:cortisol: *F*_2,396_ = 3.042, *p* = 0.049). Litter size in general negatively affected the head length (*F*_1,40_ = 4.828, *p* = 0.034).

Males generally showed a stronger and faster increase in head length compared to females (Fig. 1c), as indicated by a higher linear effect of age (Tab. 1). Nonetheless, a more strongly pronounced negative quadratic effect also indicated an earlier plateau phase in males, where no further increase was observed (Fig. 1c, Table 1). Male individuals generally exhibited a greater head length compared to females at an age of 120 days (Table 2).

Saliva cortisol concentrations negatively affected the head length of control males, while this effect was significantly diminished in PUFA males only (Fig. 1c insert graph, Table 1). However, PUFA and SFA males and all female groups remained unaffected in their head length by saliva cortisol concentrations (Fig. 1c insert graph, Table 1). A sex difference regarding this effect was therefore detected in the control group again, while no further differences occurred (Fig. 1c insert graph, Table 1).

### Testes development

**Figure 2:**
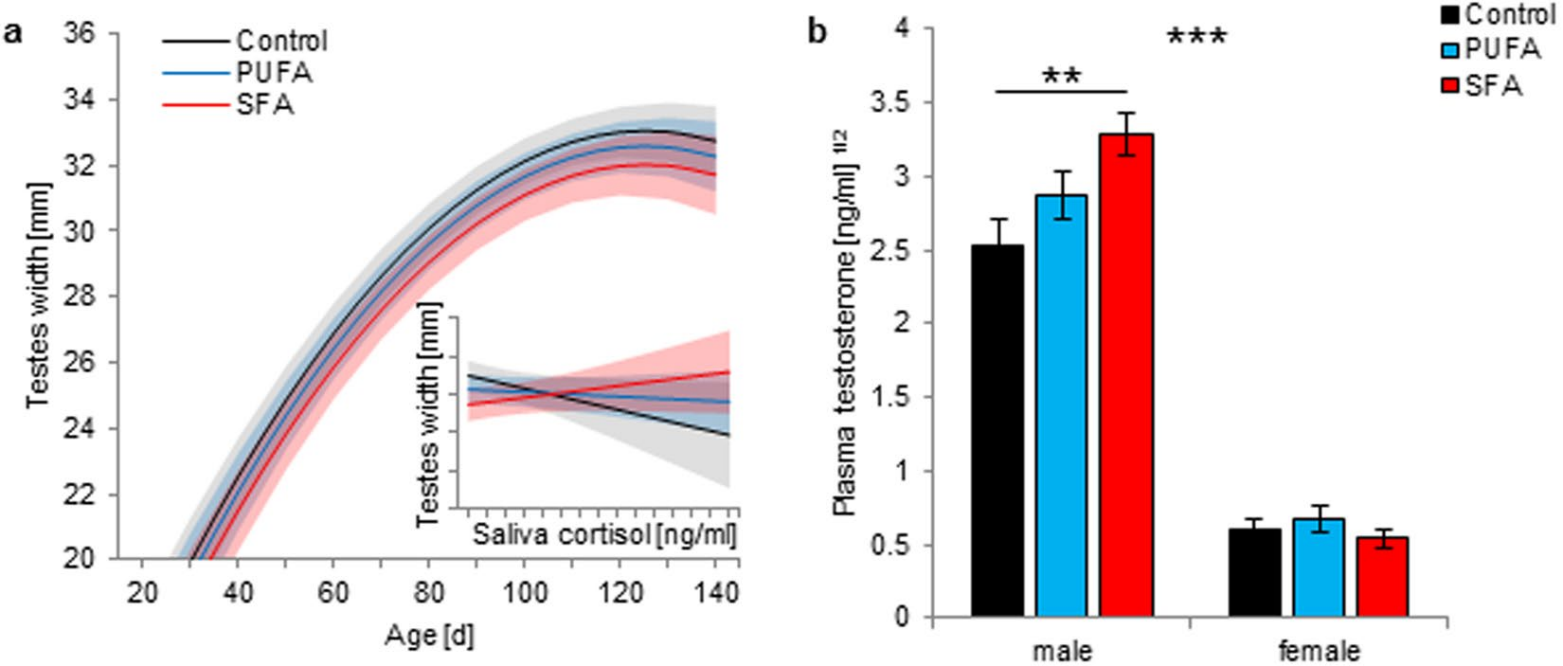
Testes development in guinea pigs maintained on a control, high-PUFA, or high-SFA diet. (**a**) Effects of age and saliva cortisol concentrations (insert graph, saliva cortisol: 0–140 ng/ml, testes width: 24–34 mm) on testes width in males (number of observations: 247). Filled areas represent the 95% confidence interval. Sample sizes: control: 14; PUFA: 14; SFA: 13. Mean number of repeated measurements: 6.02. Statistics and effect sizes are outlined in Table 1. (**b**) Plasma testosterone concentrations (means + s.e.m.) of males and females (number of observations: 193). Sample sizes: control: 14♂, 10♀; PUFA: 14♂, 9♀; SFA: 13♂, 9♀. Mean number of repeated measurements: 2.8. **p ≤ 0.01, ***p ≤ 0.001 (comparing males in females in general).

Testes width in males, as a proximate for testes size and development, was measured from the first day after their descent. Testes width increase with age (*F*_2,201_ = 217.945, *p* < 0.001) was not related to any diet effects (Fig. 2a), resulting in similar testes widths at an age of 120 days (Table 2). Cortisol showed an interaction effect with diet on testes width (diet:cortisol: *F*_2,201_ = 3.384, *p* = 0.036), and litter size in general showed a negative effect (*F*_1,19_ = 5.774, *p* = 0.027). Saliva cortisol concentrations negatively affected testes width of control males, while no such effects were detected in PUFA and SFA males. A significant difference in this effect, however, was detected between control and SFA males only, whereas the cortisol-related effect in SFA males was even slightly positively pronounced compared to controls (Fig. 2a insert graph, Table 1).

Plasma testosterone was recorded in both sexes; male levels were assumed to reflect testicular activity, female levels were considered as control values. Changes in plasma testosterone concentrations with age differed between the sexes in general (sex:age: *F*_2,120_ = 4.265, *p* = 0.016). Additionally, a highly significant interaction of diet and sex was detected (diet:sex: *F*_2,41_ = 7.866, *p* = 0.001). Testosterone concentrations in males slightly increased from 60 to 90 days of age, but decreased again until an age of 120 days; females showed no changes with age (Table 1, effect not shown), but constantly lower concentrations compared to males (Fig. 2b). Nonetheless, while plasma testosterone concentrations in females were not affected by the dietary treatments, SFA males exhibited significantly higher testosterone levels compared to control males, with intermediate levels in PUFA males (Fig. 2b). This effect remained throughout the observation period (Table 2).

### Body mass in males

In order to determine the role of the observed differences in testes development on the body mass gain in males, we re-calculated linear-mixed models on the body mass gain with age, including only data of days where plasma testosterone and testes width measurements have also been taken.

**Figure 3:**
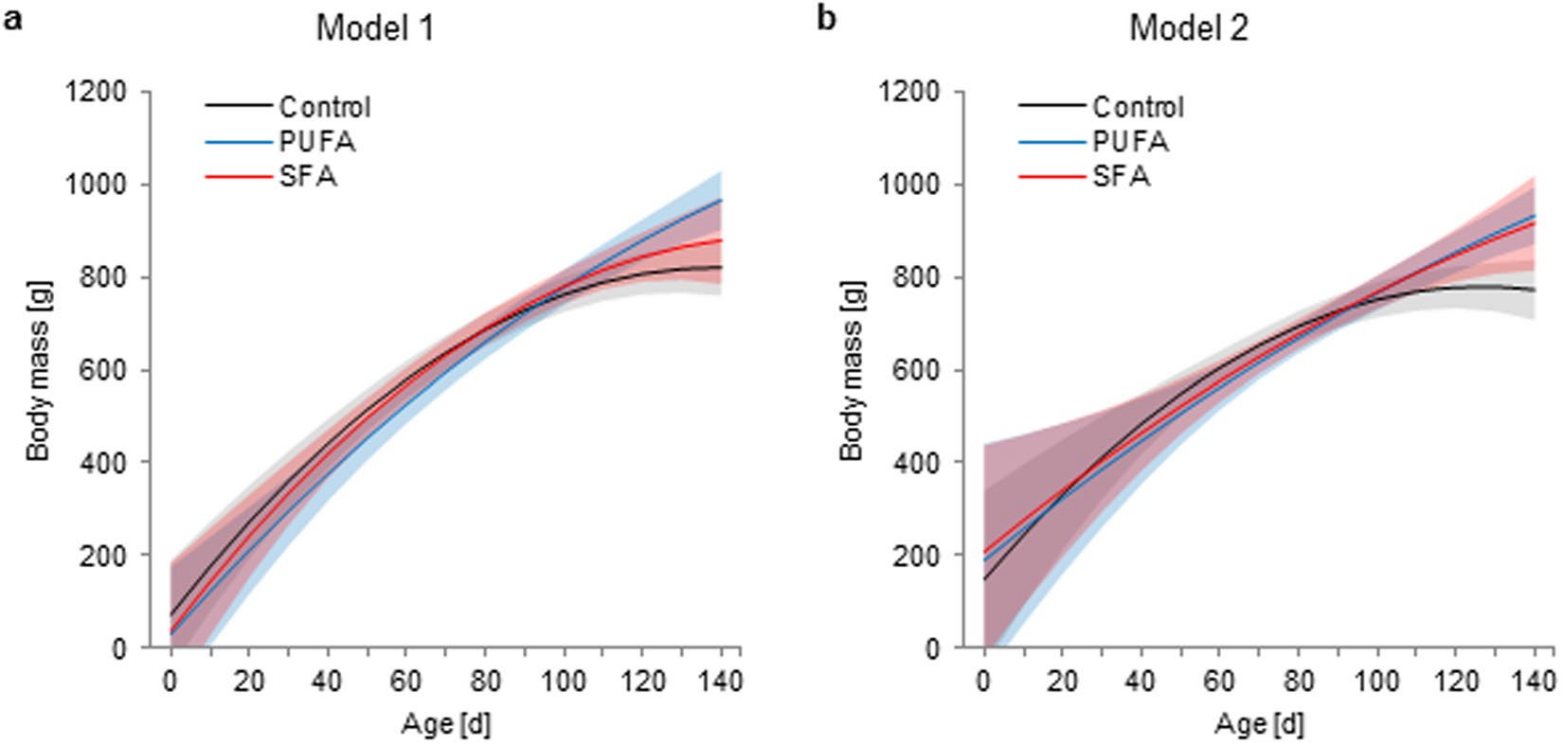
Male-specific development of body mass in guinea pigs maintained on a control, high-PUFA, or high-SFA diet. (**a**) Male-specific body mass model 1: Age-effect on body mass, corrected for saliva cortisol concentrations. (**b**) Male-specific body mass model 2: Age-effect on body mass, corrected for saliva cortisol and plasma testosterone concentrations interacting with testes width. (**a**,**b**) Filled areas represent 95% confidence intervals. Sample sizes: control: 14; PUFA: 14; SFA: 13. Mean number of repeated measurements: 2.59. Statistics and effect sizes are outlined in Table 1.

Male-specific body mass model 1: In a first step, only the effects of diet, age, and saliva cortisol, as performed in the beginning, but now with this reduced dataset on males, revealed a significant interaction of diet and age (diet:age: *F*_4,56_ = 4.704, *p* = 0.002), while a difference in the diet-specific cortisol effects missed the criterion of significance only marginally (diet:cortisol: *F*_2,56_ = 2.687, *p* = 0.077). Although differences between the linear and quadratic age effects were not significant (Table 1), PUFA males again showed the most linearly pronounced body mass gain with age, while control males reached the plateau phase earlier, and SFA males exhibited an intermediate body mass gain (Fig. 3a). Cortisol negatively affected the body mass of control males, but showed no effect on PUFA and SFA males in this reduced data set (Table 1). Nonetheless, PUFA males still exhibited the highest body mass at age 120 days; values were lowest in control and intermediate in SFA males (Table 2). As this coincides with the major body mass analysis as outlined above, this first male-specific body mass model therefore confirms the validity of the reduced dataset for males.

Male-specific body mass model 2: In a second analysis step on this reduced data set, saliva cortisol and plasma testosterone concentrations – both hormones interacting with testes width – were included as predictors. While the body mass gain with age still differed between the diets (diet:age: *F*_4,44_ = 5.005, *p* = 0.002), diet also showed interaction effects with cortisol by testes width (diet:cortisol:testes width: *F*_2,44_ = 4.834, *p* = 0.013) and testosterone by testes width (diet:testosterone:testes width: *F*_2,44_ = 3.820, *p* = 0.030). Although no straight differences were detected in any age-effect, PUFA and SFA males showed nearly the same coefficients regarding the linear and quadratic effects of age (Table 1). Accordingly, they showed the same linearly pronounced body mass gain with age and final body mass at age 120 days, whereas control males (comparable to the major body mass model and the male-specific body mass model 1) still reached the plateau phase earlier and finally remained on a lower body mass (Fig. 3b, Table 2). Additionally, a negative effect of saliva cortisol concentrations and a positive effect of plasma testosterone levels on body mass in SFA males were both diminished by their interaction with increasing testes width (Table 1). The effects of both hormones therefore attenuated with age and sexual maturity. Additionally correcting for these effects resulted in similar predicted body masses in 120-day-old PUFA and SFA males.

### Plasma fatty acids

Plasma PUFA:SFA and n-6:n-3 ratios, as the most important indicators of the plasma fatty acid status, were significantly altered by the dietary treatments and also differed between the sexes at an age of 120 days.

**Figure 4:**
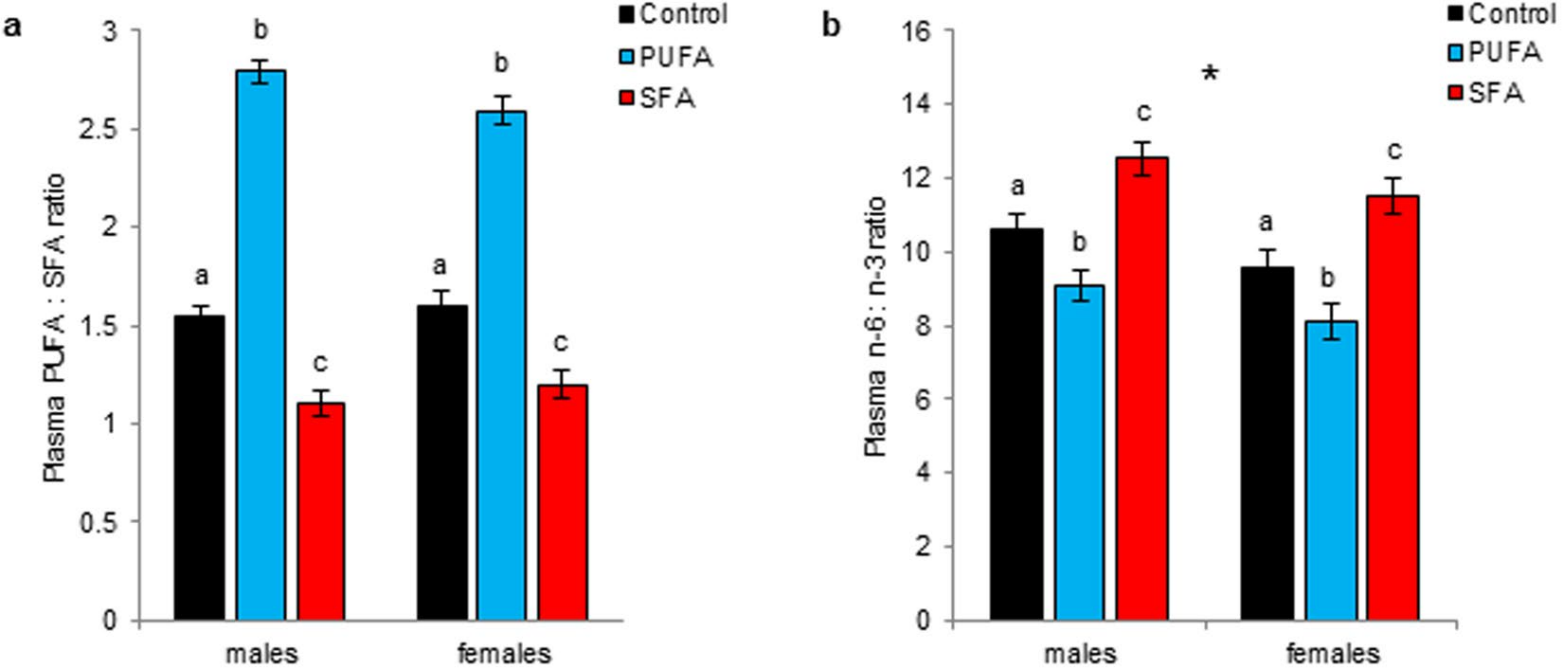
Plasma fatty acid status in male and female guinea pigs maintained on a control, high-PUFA, or high-SFA diet. (**a**) Plasma PUFA:SFA ratio (means + s.e.m.) and (**b**) plasma n-6:n-3 PUFA ratio (means + s.e.m.) at an age of 120 days. (**a**,**b**) Sample sizes: control: 14♂, 10♀; PUFA: 13♂, 9♀; SFA: 13♂, 9♀. Different superscript letters indicate significant differences between diets (*p* ≤ 0.05). *p ≤ 0.05 (comparing males and females in general).

The plasma PUFA:SFA ratio differed between the dietary groups, while sex had no effect, and the interaction of diet and sex missed the criterion of significance only marginally (diet: *F*_2,62_ = 206.072, *p* < 0.001; sex: *F*_1,62_ = 0.400, *p* = 0.530; diet:sex: *F*_2,62_ = 2.829, *p* = 0.067). PUFA animals generally showed the highest PUFA:SFA ratio (*F* = 287.512, *p* < 0.001), with slightly, but not significantly higher levels in PUFA males, while SFA animals showed the lowest ratio (*F* = 40.675, *p* < 0.001) (Fig. 4a).

The plasma n-6:n-3 ratio was significantly influenced by diet and sex (diet: *F*_2,64_ = 18.774, *p* < 0.001; sex: *F*_1,64_ = 4.583, *p* = 0.036). The interaction of diet and sex was removed from the model beforehand based on the AIC (diet:sex: *F*_2,62_ = 0.881, *p* = 0.420). The n-6:n-3 ratio was in generally higher in males than in females, but highest in SFA animals (*F* = 12.328, *p* < 0.001) and lowest in PUFA animals (*F* = 7.458, *p* = 0.008) (Fig. 4b).

### First-year survival probability

**Figure 5:**
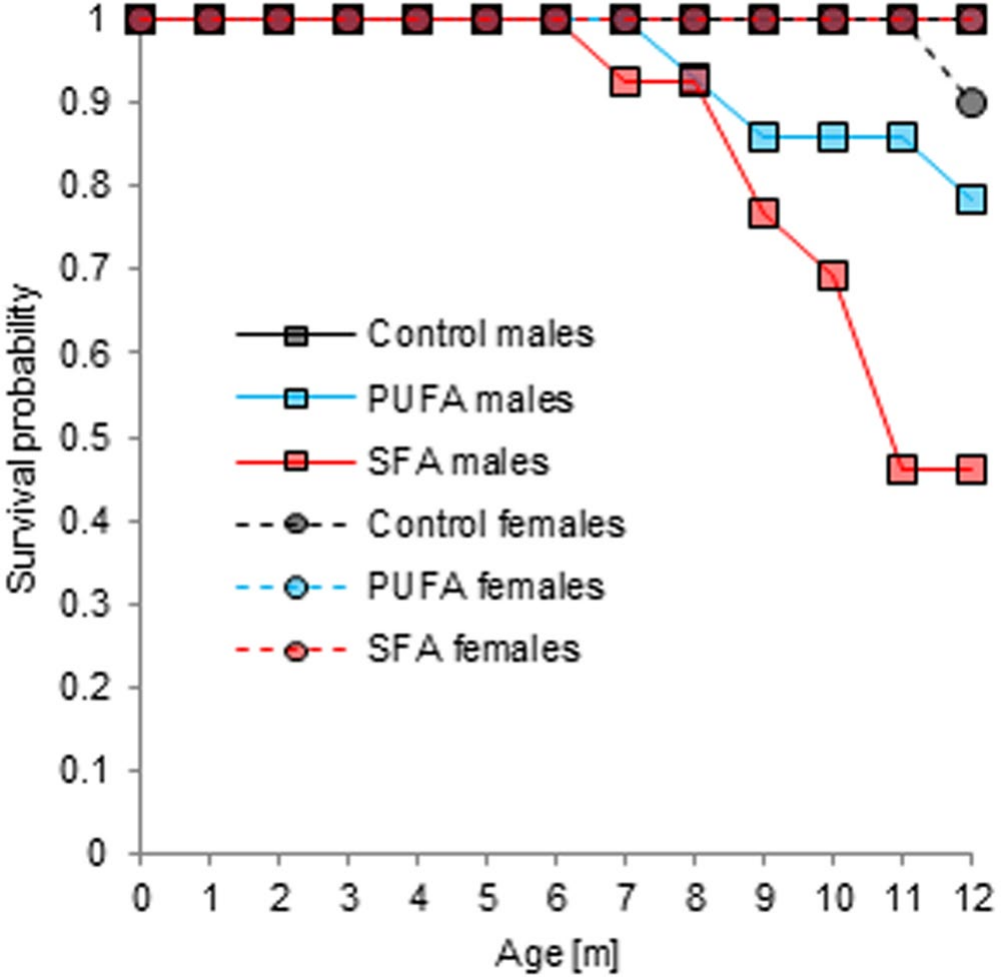
First-year survival probability by age in male and female guinea pigs maintained on a control, high-PUFA, or high-SFA diet. Sample sizes: control: 14♂, 10♀; PUFA: 14♂, 9♀; SFA: 13♂, 9♀. Note: Curves for control males, PUFA females, and SFA females run one on top of the other as they remained on a probability of 1 throughout.

The survival rate in the animals’ first 12 months of life was significantly influenced by the dietary treatments in a sex-specific manner (diet: *χ*^2^ = 13.398, *p* = 0.001; sex: *χ*^2^ = 1.447, *p* = 0.229; diet:sex: *χ*^2^ = 8.358, *p* = 0.015). All control males survived the first year, while 3 out of 14 PUFA males (=21.4%) and 7 out of 13 SFA males (=53.8%) died between 7 and 12 months of age (Fig. 5). Females, in contrast, generally showed very high survival rates within their first year of life: only 1 out of 10 control females (=10%) died at an age of approximately 12 months (Fig. 5). The probability of surviving the first 12 months of life therefore differed among males only (*χ*^2^ = 10.699, *p* = 0.005). It was significantly decreased in SFA males (PUFA males: *χ*^2^ = 1.493, *p* = 0.222; SFA males: *χ*^2^ = 7.566, *p* = 0.006), whereas no differences were detected among females (*χ*^2^ = 1.867, *p* = 0.393).

## Discussion

We demonstrated that pre- and postnatal supplementations with PUFAs or SFAs differently modulate structural growth rates and adult outcomes in a sex-specific relation to saliva cortisol secretion rates in preadolescent male and female guinea pigs. Dietary PUFA and SFA supplementations both resulted in a more linearly pronounced body mass gain in males and females compared to control animals. Control animals, in contrast, showed a much stronger body mass increase during the first two months of life and reached their plateau phase earlier, with no further changes in their body mass from approximately 100 days of age. Moreover, head length in both sexes and testes width in males showed similar changes with age, but the development of these structures did not differ between the dietary treatments. This generally demonstrates the fast postnatal development in this precocial species, which is related to a higher energetic effort compared to altricial species^30^ and has previously been shown to be improved by high energetic diets^26^. It has to be noted, however, that a too fast postnatal growth in humans caused by low birth weights or increased postnatal energy intakes could also impair metabolic processes such as the insulin sensitivity and increase the risk for obesity^31,32^. Likewise, high dietary fat intake increases an organism’s body mass gain, presumably because of more pronounced abdominal fat stores, but this effect and related impact on metabolic processes may emerge sex-specifically, as recently shown in rats^33^. Although this may similarly apply to PUFAs and SFAs, findings in rats, pigs, and broiler chickens clearly demonstrate a preferred mobilization of PUFAs from fat stores and increased beta-oxidation rates^18,34,35^. This perhaps results in a higher energy turnover and a redirection of the available energy from abdominal fat stores in physiological processes. This may further be promoted by the anti-inflammatory actions of n-3 PUFAs and their positive effect on hypothalamic insulin resistance and whole body energy balance^10^. Translated to our findings, dietary PUFAs may have reduced body mass gain in the beginning, probably due to higher oxidation rates, but positively affected the energy turnover throughout the experiment. This resulted in an undisturbed and linear pronounced body mass gain and improved body conditions with age. Control animals, in turn, perhaps could no longer maintain the positive energy balance due to the higher demands with age and body size.

Skeletal muscles are suggested to preferably benefit from the available energy via enhanced fatty acid oxidation^18,36^, whereas the brain, for example, primarily utilizes glucose as an energy source^37^. PUFAs apparently protect proteins and therefore muscle mass during stressful and energetically demanding periods, perhaps resulting from altered insulin signaling^36^. This would be important because we detected a very strong negative effect of saliva cortisol concentrations on the growth of control males, including on body mass, head length, and testes width, which was significantly diminished especially by PUFAs. Therefore, elevated glucocorticoid secretion rates during energetically demanding situations and periods could severely impact the postnatal structural development of male guinea pigs. PUFA intakes, in contrast, may maintain body conditions during such events, perhaps reflecting improved metabolic processes and enhanced fatty acid oxidation rates^38^. PUFAs may therefore also compensate the higher energetic demands in developing males, as indicated by increasing cortisol levels with age, and uncouple them from the comprehensive cortisol actions on metabolic processes and gluconeogenesis^5^. This interpretation implies that internal energy reserves are no longer required for growth and maturation processes, which results in an undisturbed development and highest body conditions in adulthood.

In contrast to PUFAs, SFAs are deposited in abdominal fat stores rather than being oxidized, yet without apparent effects on body mass^18,39^. Indeed, SFA males in our study showed a similar body mass gain as PUFA males in the first 60–80 days of life. Their body mass, however, rather tended to reach the plateau phase at an age of 120 days, when body mass in PUFA males still increased. Our analyses of testes width in males and the related plasma testosterone concentrations suggest that the limited body mass gain in SFA males was caused more by a redirection of available energy in testicular functions than simply by their highly increased saliva cortisol concentrations. Although testes width did not differ between the dietary groups, saliva cortisol concentrations showed a slightly pronounced positive effect on testes width in SFA males, which differed significantly from control males. This effect and significantly increased plasma testosterone concentrations might indicate an energetic investment in testes development in SFA males. In general, the development of reproductive organs, reproductive performance, and therefore lifetime reproductive success are closely linked to the energy balance of an individual^40^. A redirection of available energy from dietary SFAs, which was indicated by the highly increased cortisol levels, might be important to ensure normal testes development in these animals, which would otherwise be promoted by n-3 PUFAs^41^. Increased dietary SFA intake and a lower PUFA:SFA ratio reduces the overall availability of PUFAs for metabolic processes^17^ and for promoting the biochemical properties of neuronal cell membranes and HPA- and hypothalamic-pituitary-gonadal (HPG)-axis functions^20^. N-3 and n-6 PUFAs are important but opposed regulators of key enzymes involved in steroidogenesis and in the translocation of cholesterol^20^. SFAs, in contrast, elevate plasma low-density-lipoprotein cholesterol levels in guinea pigs^42^, which may be primarily used for steroid hormone synthesis, at least in humans^43^. As guinea pigs represent a model species regarding the lipid and cholesterol metabolism in humans^19^, similar relations can be assumed. A too low PUFA:SFA ratio and a high n-6:n-3 ratio, as found in the plasma of 120-day-old SFA males, could have negatively affected the regulation of these processes and steroidogenesis. This, in turn, would result in excessive HPA- and HPG-axis activities and impaired homeostasis due to the necessary investment in testes development. As plasma testosterone levels in females were, in contrast to males, not affected by dietary SFAs, a male-specific effect on steroidogenesis, probably resulting from testicular activity, seems most reasonable.

Finally, the assumed energetic investment via cortisol actions towards testes development and testicular functions in SFA males also explained the observed difference in the body mass gain versus PUFA males. The first calculated model (male-specific body mass model 1) on the reduced dataset for the body mass gain in males yielded the same results as analyses on the complete dataset (see Tables 1 and 2). This first step basically confirmed the validity of the reduced dataset. Correcting for the postulated investment in testes development and the elevated testosterone levels (male-specific body mass model 2) extinguished the difference between PUFA and SFA males in body mass gain and predicted body mass at 120 days. This indicates that PUFAs and SFAs usually provided the animals with the same energy content of 38.9 kJ or 9.3 kcal per gram during juvenile development. Nonetheless, intakes of these nutrients as 10% (w/w) of the whole diet clearly resulted in different energy accumulations and HPA- and HPG-axis activities in relation to hormone-related effects. These processes ultimately resulted in the detected diverse developments in males. While testosterone is well-known as the major androgen involved in the development of males and highly coincides with testes development in guinea pigs^44^, cortisol usually negatively affects body conditions^12,17^. The male-specific body mass model 2 also revealed a positive effect of testosterone and a negative effect of cortisol on body mass in SFA males, but both effects were statistically diminished by their interactions with testes width. This would reflect an obvious trade-off between body conditions and gonadal functions, which is negatively affected by an impaired homeostasis and energy balance^40^. Although the hormone-related effects in SFA males disappeared with the completion of the gonadal maturation process, dietary SFAs and unbalanced PUFA:SFA ratios seem to contribute to or even boost this trade-off during juvenile development.

Although increased cortisol secretion rates in SFA males were apparently important for testes development, the pathologies related to long-term allostatic processes cannot be neglected^45^. Increased steroid hormone secretion rates during the postnatal development in SFA males were followed by a significantly higher mortality rate during adulthood. N-3 PUFA metabolites such as eicosanoids^46^ and glucocorticoids in general^47^ exert anti-inflammatory actions, whereas n-3 PUFA deficiencies and HPA-axis dysfunctions may profoundly impair the immune system and inflammatory responses^48,49^. Although the cause of deaths in SFA males was not investigated in this study, their highly increased cortisol concentrations may indicate a detrimental chronic stress state^49,50^. This is usually accompanied by impaired glucocorticoid receptor sensitivity or even a resistance to cortisol, which is suggested to negatively affect HPA-axis responses to immune challenges, impair homeostasis, and increase the disease risk^49,51,52^. SFA males in our study were presumably unable to respond adequately to any physiological perturbations, possibly promoting a fast progress of pathologies and early death. These results highlight the possible long-term consequences of increased steroid hormone concentrations and the importance of adequate dietary PUFA intake in balancing body homeostasis.

In contrast to males, female individuals were not markedly affected in their postnatal development by dietary fatty acid intakes. This may reflect a general sexual dimorphism in the progress of maturation, body conditions, and different steroid hormone secretion rates^22,23^, but perhaps also a different n-3 PUFA metabolism^53^. Females perhaps face lower energetic needs during their postnatal development, an interpretation supported by the constant or decreasing cortisol concentrations with age and the lack of cortisol-related effects in our study. Nonetheless, adequate energy and PUFA intakes are definitely also required for ovarian development and female reproductive functions in general^54^. Females additionally seem to exhibit a higher enzymatic capacity regarding the accumulation and/or elongation of n-3 PUFAs, whereas males are more dependent on adequate dietary intakes but might also show higher oxidation rates of these nutrients^53,55^. The latter effect is indicated by higher plasma n-6:n-3 ratios in the males studied here, resulting from lower plasma n-3 PUFA levels. This would make dietary n-3 PUFA levels more important in male individuals to ensure optimal neurophysiological or HPA-axis functions and to provide sufficient energy via preferential n-3 PUFA oxidation rates. Lower energetic requirements and higher n-3 PUFA accumulations in females perhaps also buffered homeostatic perturbations, for example increased saliva cortisol concentrations caused by high SFA intakes. We therefore conclude that a naturally occurring or diet-induced high n-3 PUFA status positively affects HPA-axis functions and physiological stress responses, which enables an undisturbed postnatal development.

Our results demonstrate that dietary PUFAs and SFAs sex-specifically modulate the postnatal structural development in guinea pigs. This is associated with their saliva cortisol secretion rates as mediators of an individual’s homeostasis. Males seem to be more susceptible to inadequate PUFA supplies, perturbations of homeostasis caused by elevated SFA levels, or increased cortisol concentrations in general, resulting in different patterns of their structural development. We therefore postulate that the outstanding positive effects of PUFAs on HPA-axis functions and glucocorticoid-effects on metabolic processes play a major role in postnatal development. This highlights the high biological relevance of these nutrients until adulthood. Note in this context that the social environment could also play a fundamental role in early developmental processes, especially when considering effects on the endocrine system, as has been repeatedly shown in guinea pigs^56–58^. The animals in our study were kept in social groups during their postnatal periods, and first analyses indicate an increased aggressiveness in SFA males. This, however, need not necessarily be related to steroid hormone secretion rates in response to dietary SFAs, as we showed recently^17^. Additionally, the social context would definitely not explain the relations between steroid hormones and the structural growth we found here. Nevertheless, also with regard to lowered SFA and cortisol concentrations, adequate PUFA supplies during early developmental periods represent crucial nutritional influences on fetal and/or developmental programming and for adult mental and metabolic health^2,14^, which are now revealed to possibly be sex-specific.

## Methods

### Ethical statement

Experiments were performed in adherence with EU guidelines for the protection of animals used for scientific purposes (Directive 2010/63/EU) and have been examined and approved by the animal welfare committee of the Faculty of Life Sciences, University of Vienna (# 2014-005), and the Austrian Federal Ministry of Science and Research (BMWF-66.006/0024-II/3b/2013).

### Animals and experimental diets

All domestic guinea pigs used in this study belonged to a heterogeneous stock of animals established in 2013 at our department. All involved animals could be individually identified by natural fur colorations, were sexually intact, and accustomed to daily contact with humans. Sixty animals (30 males and 30 females; mean age: 21.1 ± 1.2 months; mean body mass: 805 ± 20 g) were randomly allocated to single-sexed social groups of ten individuals, which was carried out by persons that were blind to the planned study. This yielded three male and three female groups, which were housed in separate, but equally equipped enclosures (2 m × 1.6 m each). Each enclosure was environmentally enriched with different-sized shelters and platforms and the floor was covered with bedding material. Water was always available ad libitum in several drinking bottles. Daily provided food before the experiment started consisted of ad libitum guinea pig pellets (ssniff V2233, ssniff Spezialdiäten GmbH, Soest, Germany) and 50 g of hay per group to promote tooth abrasion and support the digestive system of the animals. A temperature of 20 ± 2 °C, a humidity of 50 ± 5%, and a light-dark cycle of 12 h each (lights on at 07:00 a.m.) were maintained throughout the study.

Each socially established single-sexed group was randomly subjected to one of three different diets (one male and one female group per diet): a control, high-PUFA, or high-SFA diet. The investigators were aware of the group allocation throughout the study. For the control diet, pure pellets were provided, while pellets were enriched in either 10% (w/w) walnut oil (99.6% fat content; 73.9% total PUFAs, 9.9% total SFAs) in case of the high-PUFA diet or in 10% (w/w) coconut fat (100% fat content; 1.5% total PUFAs, 92% total SFAs) in case of the high-SFA diet. This resulted in highly different fatty acid compositions and fat contents of the experimental diets (Table 3). After 100 days on the respective dietary regime, male and female groups maintained on the same diet were combined for mating. Once pregnancy was noted in females, single-sexed groups were established again. Feeding procedures continued during the mating phase, as well as in females during subsequent gestation and lactation. A total of 69 pups (control: 14♂, 10♀; PUFA: 14♂, 9♀; SFA: 13♂, 9♀) from 25 litters (control: 8; PUFA: 7; SFA: 10) were born alive and housed from birth in the same social group as their mothers, together with the remaining pregnant and/or lactating females and pups of the other litters, and maintained on the same diets. As guinea pigs are highly precocial, the provided diet was ingested by the pups from 2–3 days after birth, although pups were also lactated until an age of approximately 15–20 days. At 20–30 days of age, pups were separated from their mothers and integrated into new single-sexed social groups containing only animals of the same generation, sex, and diet. Relatedness as well as litter size were considered in the analyses, while no effects of the dietary treatments on the individual pup’s birth mass were found^29^ and birth mass was therefore not included in the analyses.

**Table 3:** Fatty acid composition and fat content of the experimental diets.

Fatty Acid	Experimental diet					
	Fatty acid composition (% of total fat)^a^			Fatty acid content (% of total food)^b^		
	Control	PUFA	SFA	Control	PUFA	SFA
12:0	0.00	0.00	37.11	0.00	0.00	4.81
14:0	0.61	0.00	17.13	0.02	0.00	2.22
16:0	16.16	9.00	13.01	0.53	1.16	1.69
18:0	3.35	2.92	4.32	0.11	0.38	0.56
20:0	0.30	0.00	0.00	0.01	0.00	0.00
22:0	0.00	0.00	0.13	0.00	0.00	0.02
Total SFA	20.43	11.93	71.70	0.67	1.54	9.30
15:1	0.00	0.00	0.11	0.00	0.00	0.01
16:1	0.61	0.00	0.00	0.02	0.00	0.00
18:1 n-7	0.00	0.96	0.40	0.00	0.12	0.05
18:1 n-9	18.90	15.41	9.85	0.62	1.99	1.28
Total MUFA	19.51	15.41	9.96	0.64	1.99	1.29
18:2 n-6	50.00	60.57	15.66	1.65	7.84	2.03
18:3 n-6	0.00	0.30	0.20	0.00	0.04	0.03
18:3 n-3	10.06	10.83	2.08	0.33	1.40	0.27
Total PUFA	60.06	71.70	17.94	1.98	9.27	2.33
n-6: n-3 ratio	4.97	5.62	7.64	4.97	5.62	7.64
PUFA: SFA ratio	2.94	6.01	0.25	2.94	6.01	0.25
Total fat	100.00	100.00	100.00	3.30	12.93	12.97

^a^Based on gas chromatography analyses of the experimental diets.

^b^Based on calculations combining the manufacturer information for guinea pig pellets (ssniff V2233, ssniff Spezialdiäten GmbH, Soest, Germany) and the fatty acid analyses of the experimental diets using gas chromatography.

### Experimental procedure

After the lactation period, lasting until an age of 15–20 days, saliva samples were collected biweekly until an age of approximately 120 days; all pups were weighed and their head lengths, as well as testes width in males, measured on the same days. This was done to document individual saliva cortisol secretion rates as an indicator of the animals’ homeostasis and their effects on the postnatal juvenile developmental period. An additional saliva sample per animal was collected directly after the separation from the mother and the integration into the new single-sexed social groups. This was done in order to include this perhaps stressful situation and its potential effects on development in the analyses. Animals were weighed on a digital kitchen balance (accuracy: ± 1 g). The head length, from the occipital bone to the tip of the nose, and testes width in males were measured using a digital caliper rule (accuracy: ± 0.1 mm). Saliva samples were collected by inserting a standard cotton bud into the animal’s mouth and gently collecting saliva from inside the cheeks for approximately one minute^59^. After centrifugation (14000 rpm, 10 min), pure saliva was stored at −20 °C until analysis of saliva cortisol concentrations. All measurements were performed from 09:00 a.m. until 11:00 a.m.

The above-mentioned measurements were started after the lactation period in order to exclude lactation as a possible bias on juvenile development due to individually different durations of the lactation period and allolactation which occurred in some animals of each group. Saliva cortisol concentrations and the postnatal development in this precocial species were therefore assumed to be unbiased by irregular maternal behaviour for the first time after the short lactation period and therefore solely influenced by the different dietary treatments. As the structural growth in guinea pigs usually reaches a plateau phase at an age of 120 days, where no further significant changes may be observed, this age was set as a proximate for the end of the juvenile developmental phase. The chosen time frame also corresponded to previous documentations of the juvenile development in guinea pigs^23,26^.

We additionally decided to perform the measurements on specified calendar days rather than on an individual’s age basis. This, on one hand, enabled controlling for possible disturbances because all animals were housed in their single-sexed groups in the same room and would have been similarly affected by any external disturbances. On the other hand, this yielded a higher variability of age-specific measurements across the animals and therefore a continuous distribution of the animals’ age and the related age-specific measurements. Therefore the individual age (in days) was noted at each measurement in order to include this as a continuous variable in the statistical analyses.

Additionally, plasma testosterone concentrations were measured in blood samples collected from marginal ear veins at mean ages of 60, 90, and 120 days. Approximately 200 µl of whole-blood was collected with heparinized (5000 U) micropipettes after puncturing prominent ear veins^59^. Plasma was separated by two consecutive centrifugation steps (14000 rpm, 10 min) and pure plasma samples were stored at −20 °C until further analysis. After the last measurements at an age of approximately 120–140 days, additional blood samples were collected in order to determine plasma fatty acid patterns at the end of the juvenile developmental period (as an indicator of the integration of these molecules in the metabolism during the juvenile developmental period).

Deaths of single animals were recorded within the first 12 months of life, even after all measurements regarding the juvenile developmental period were completed (note: no animal died during the juvenile developmental period before 6 months of age). This was done to determine possible effects of any early postnatal perturbations on the probability of surviving maturation and early adulthood.

### Hormone analyses

Hormone concentrations were analyzed by biotin-strepdavidin enzyme-linked immunoassays using cortisol- and testosterone-specific antibodies as described previously^60,61^. Both antibodies are biologically valid regarding adequate hormone analyses in guinea pigs^59,62^. Based on the previous determination of the 50% binding of diluted pooled samples, single saliva samples were diluted 1:50 and cortisol concentrations measured in 10 µl inputs. Intra- and interassay coefficients of variance were 10.29% and 4.56%, respectively. Plasma steroids were extracted by adding 2 ml diethylether to 100 µl pure plasma, shaking the samples four times for 15 min, and freezing them overnight at −20 °C. The non-frozen supernatants, containing the steroids, were evaporated at 30 °C, diluted 1:5 and testosterone concentrations measured in 10 µl inputs. Intra- and interassay coefficients of variance were 10.89% and 1.62%, respectively. All analyses were run in duplicates.

### Plasma fatty acid analyses

Preparation of fatty acid methyl esters (FAMEs) followed previously described protocols^12^. In brief, 35 µl plasma were transesterificated using 1 ml methanolic NaOH, containing butylated hydroxytoluene, and FAMES were obtained by adding 1 ml boron-trifluoride. FAMEs were extracted by adding 500 µl hexane four times, evaporated at 40 °C under nitrogen, and redissolved in hexane again. Separation and analysis of FAMEs was performed using an Auto-System-Gaschromatograph with flame ionization detector (Perkin Elmer, USA), equipped with a Rtx-2330 30 m × 0.25 mm × 0.20 µm silica column, using helium as a carrier gas. One µl of prepared samples was injected at 250 °C and detected at 275 °C. Single fatty acids were identified based on a 37 component FAME Mix Standard (Supelco, Bellafonte, USA) and peaks integrated using TotalChrome Workstation 6.3.0 (PE Nelson, Perkin Elmer, USA). Single fatty acids were expressed as percentage of total plasma fatty acids.

### Statistics

The courses of saliva cortisol concentrations, body mass, head length, testes width in males, and plasma testosterone levels during the juvenile developmental period were analyzed using mixed effects models. This method was used because mixed effects models enable corrections for multiple random effects and specification of the variance function of the performed analyses. ‘Diet’ (control, PUFA, SFA), ‘sex’ (female, male), and ‘age’ (continuous variable in days), as well as their 2- and 3-way interactions, were included as predictor variables in all models, except for ‘sex’ in the case of testes width. ‘Age’ was included as a second-order polynomial due to non-linear changes in the response variables. ‘Litter size’ (continuous variable 1–5) and its 2- and 3-way interactions with ‘diet’ and ‘sex’ was additionally included in order to correct for any litter size effects on the above mentioned response variables. Effects of saliva cortisol concentrations on body mass, head length, and testes width were analyzed by including ‘cortisol’ and its 2- and 3-way interactions with ‘diet’ and ‘sex’ as covariates in the models on body mass and head length. To correct for repeated measurements and relationships between single individuals, ‘pup ID’ within ‘mother ID’ was included as random intercept and ‘age’ as random slope to allow individual changes in the response variables with age. Due to heteroscedasticity of model residuals, models were corrected for different variance structures in the ‘diet:sex:age’-interaction. Maximum iterations were allowed for calculating model statistics.

In order to unravel possible effects of the observed differences in testes width development and plasma testosterone concentrations on the body mass gain in males, two mixed effects models were calculated on male body mass changes. These models included body mass data as response variables of specific days where corresponding measurements of saliva cortisol, plasma testosterone, and testes width were made. The first model included only ‘diet’, ‘age’ (as second-order polynomial), ‘cortisol’, and ‘litter size’ as predictors (interactions as outlined above), while in the second model ‘testosterone’ and ‘testes width’ were additionally included in interactions with ‘cortisol’, in order to extract the predicted body mass gain after correcting for these male-specific effects. Random effects and further specifications correspond to the models outlined above.

Plasma fatty acids were analyzed by a two-way analysis of variance (ANOVAs), including ‘diet’ and ‘sex’, on the plasma PUFA:SFA and the n-6:n-3 ratio. The survival probability by age was analysed by applying a survival analysis based on the animal’s age at death, with all animals surviving the first year of life being censored. Sex-specific chi-squared tests were applied to determine the deviations from equality of proportions (dead vs. alive) among the diets within the first year of life.

Model assumptions were checked by performing Shapiro-Wilk normality tests and Levene’s test for homogeneity of variance as well as by plotting model residuals and fitted values. To meet the model assumptions, saliva cortisol and plasma testosterone concentrations had to be transformed as response variables by applying the third and second root, respectively. Model residuals revealed no outliers and therefore all animals and measurements were included in the analyses. As the available number of animals was dependent on the reproductive success, sample sizes could not be directly controlled by the investigators. Power analysis, however, revealed that sample sizes per group were large enough in order to obtain adequate effect sizes (Cohen’s f > 0.38). Model fitting was based on the Akaike information criterion (AIC), with only relevant predictor terms and interactions remaining in the final models. Model statistics are based on type 3 sum of squares, and, therefore, results (F- and p-values) were corrected for each effect in the model. The significance level was set at *p* ≤ 0.05; all tests were two-sided. Only statistics for the highest relevant significant interactions and main effects are outlined in the Result section; for full ANOVA tables of fitted models see Supplementary Table S1. Mean effect sizes and confidence intervals, respectively standard errors, were plotted to visualize the detected effects based on the calculated models. Additional plots of raw data are provided in Supplementary Figures S1–S4.

### Data availability

The datasets generated and/or analysed during this study are available from the corresponding author on reasonable request.

## Electronic supplementary material

Supplementary Figure S1-S4 and Supplementary Table S1
