# Supplementary material for: Dietary fatty acids sex-specifically modulate guinea pig postnatal development via cortisol concentrations
Source: Sci Rep. 2018 Jan 11;8:471. doi: 10.1038/s41598-017-18978-4 (PMC5765112; doi:10.1038/s41598-017-18978-4)
Supplement: Supplementary file 1 — Supplementary Figure S1-S4 and Supplementary Table S1 [file 41598_2017_18978_MOESM1_ESM.doc]

**Dietary fatty acids sex-specifically modulate guinea pig postnatal development via cortisol concentrations**

Matthias Nemeth, Eva Millesi, Daniela Schuster, Ruth Quint, Karl-Heinz Wagner, Bernard Wallner


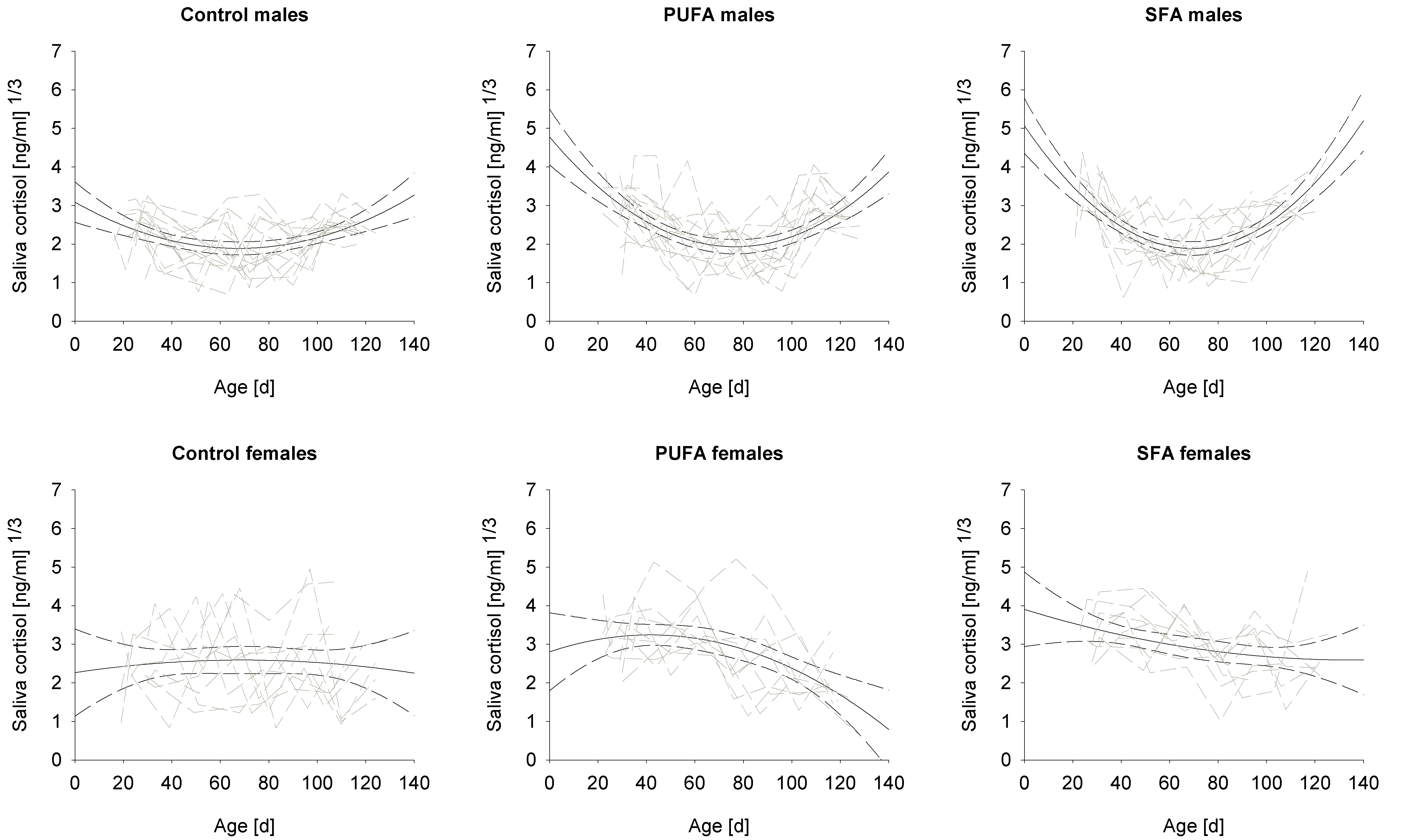


**Supplementary Figure S1** Raw data, mean effects, and 95 % confidence intervals for saliva cortisol concentrations (third-root transformed) of male and female guinea pigs maintained on a control, high-PUFA, or high-SFA diet. Each grey dashed line represents a single individual.


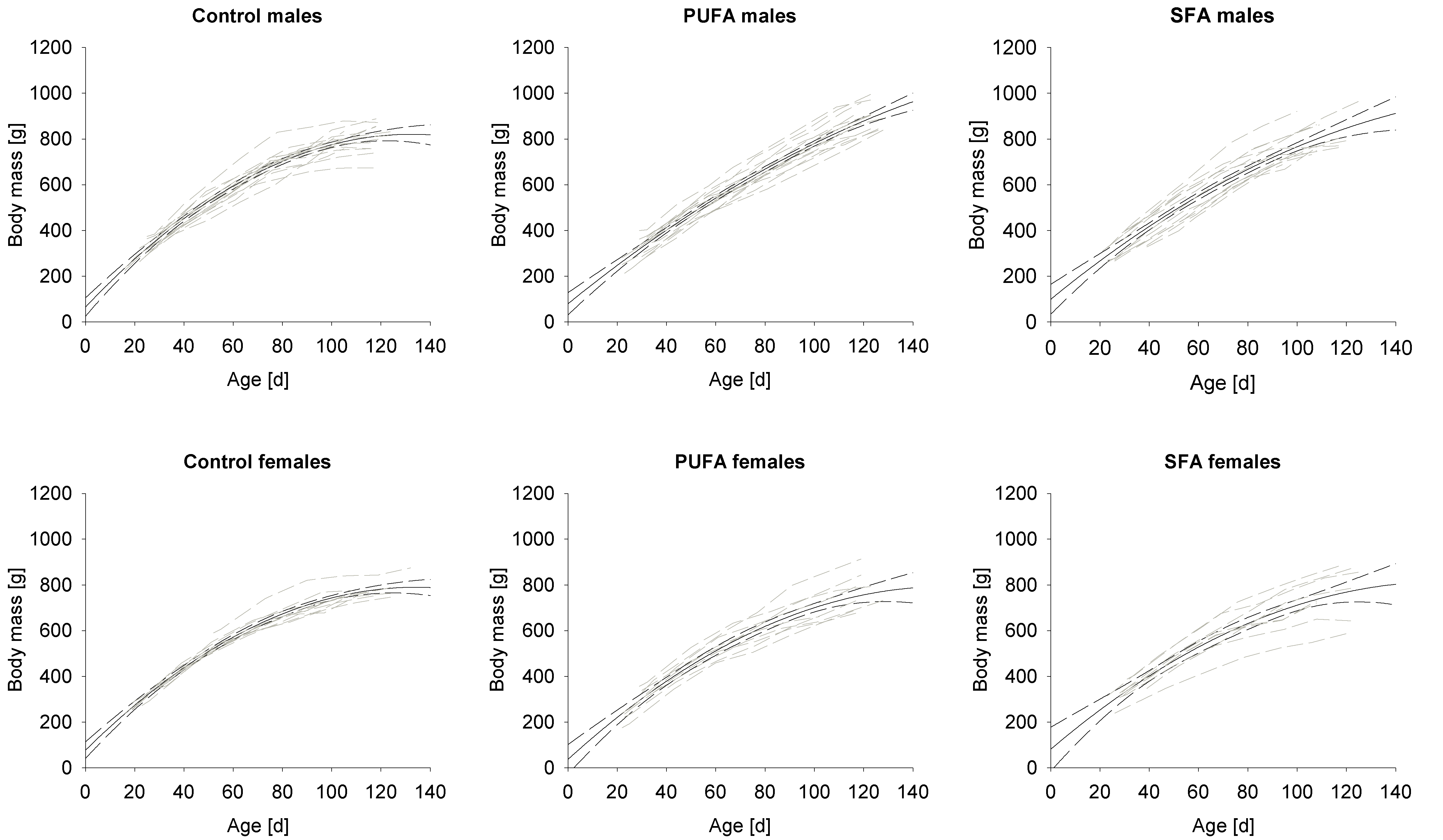


**Supplementary Figure S2** Raw data, mean effects, and 95 % confidence intervals for body mass of male and female guinea pigs maintained on a control, high-PUFA, or high-SFA diet. Each grey dashed line represents a single individual.


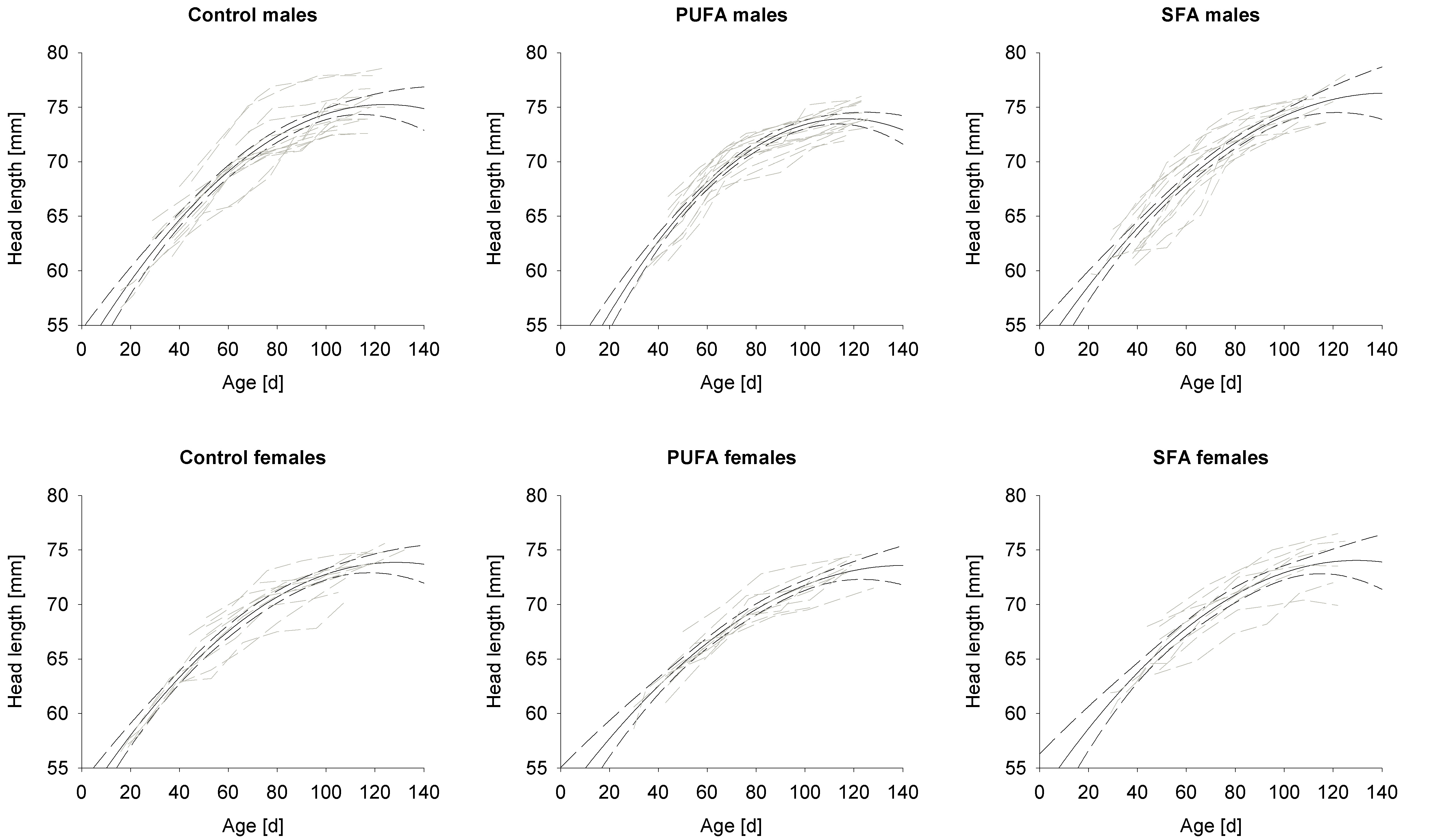


**Supplementary Figure S3** Raw data, mean effects, and 95 % confidence intervals for head length of male and female guinea pigs maintained on a control, high-PUFA, or high-SFA diet. Each grey dashed line represents a single individual.

**Supplementary Figure S4** Raw data, mean effects, and 95 % confidence intervals for testes width of male guinea pigs maintained on a control, high-PUFA, or high-SFA diet. Each grey dashed line represents a single individual.

**Supplementary Table S1** ANOVA tables for fitted models on the measured parameters in male and female guinea pigs maintained on a control, high-PUFA, or high-SFA diet.

| **Response variable** | **Predictor** | **Statistics** |  |  |
| --- | --- | --- | --- | --- |
|  |  | **df** | **F-value** | **p-value** |
| Saliva cortisol | Diet | 2,22 | 15.893 | 0.0001 |
|  | Sex | 1,43 | 15.338 | 0.0003 |
|  | Age | 2,484 | 17.294 | <0.0001 |
|  | Diet : Age | 4,484 | 7.531 | <0.0001 |
|  | Sex : Age | 2,484 | 36.231 | <0.0001 |
| Body mass | Diet | 2,22 | 1.694 | 0.207 |
|  | Sex | 1,40 | 0.028 | 0.8686 |
|  | Age | 2,474 | 450.180 | <0.0001 |
|  | Cortisol | 1,474 | 13.236 | 0.0003 |
|  | Litter Size | 1,40 | 13.430 | 0.0007 |
|  | Diet : Sex | 2,40 | 0.781 | 0.465 |
|  | Diet : Age | 4,474 | 30.286 | <0.0001 |
|  | Diet : Cortisol | 2,474 | 4.792 | 0.0087 |
|  | Sex : Age | 2,474 | 1.045 | 0.3524 |
|  | Sex : Cortisol | 1,474 | 10.149 | 0.0015 |
|  | Diet : Sex : Age | 4,474 | 6.237 | 0.0001 |
|  | Diet : Sex : Cortisol | 2,474 | 3.701 | 0.0254 |
| Head length | Diet | 2,22 | 2.043 | 0.1536 |
|  | Sex | 1,40 | 3.312 | 0.0763 |
|  | Age | 2,396 | 768.806 | <0.0001 |
|  | Cortisol | 1,396 | 7.040 | 0.0083 |
|  | Litter Size | 1,40 | 4.828 | 0.0339 |
|  | Diet : Sex | 2,40 | 0.626 | 0.5398 |
|  | Diet : Cortisol | 2,396 | 3.375 | 0.0352 |
|  | Sex : Age | 2,396 | 10.401 | <0.0001 |
|  | Sex : Cortisol | 1,396 | 4.053 | 0.0448 |
|  | Diet : Sex : Cortisol | 2,396 | 3.042 | 0.0489 |
| Testes width | Diet | 2,19 | 3.257 | 0.0608 |
|  | Age | 2,201 | 217.945 | <0.0001 |
|  | Cortisol | 1,201 | 4.378 | 0.0377 |
|  | Litter Size | 1,19 | 5.774 | 0.0266 |
|  | Diet : Cortisol | 2,201 | 3.384 | 0.0359 |
| Plasma testosterone | Diet | 2,22 | 7.390 | 0.0035 |
|  | Sex | 1,41 | 1.863 | 0.1797 |
|  | Age | 2,120 | 5.435 | 0.0055 |
|  | Diet : Sex | 2,41 | 7.866 | 0.0013 |
|  | Sex : Age | 2,120 | 4.265 | 0.0162 |
| Male body mass | Diet | 2,20 | 0.228 | 0.7979 |
| model 1 | Age | 2,56 | 73.823 | <0.0001 |
|  | Cortisol | 1 | 9.653 | 0.003 |
|  | Diet : Age | 4,56 | 4.704 | 0.0024 |
|  | Diet : Cortisol | 2,56 | 2.687 | 0.0769 |
| Male body mass | Diet | 2,20 | 0.204 | 0.8174 |
| model 2 | Age | 2,44 | 26.199 | <0.0001 |
|  | Cortisol | 1,44 | 0.025 | 0.8762 |
|  | Testosterone | 1,44 | 0.644 | 0.4267 |
|  | Testes width | 1,44 | 2.425 | 0.1266 |
|  | Cortisol : Testes width | 1,44 | 0.003 | 0.9554 |
|  | Testosterone : Testes width | 1,44 | 1.096 | 0.3008 |
|  | Diet : Age | 4,44 | 5.005 | 0.0021 |
|  | Diet : Cortisol | 2,44 | 5.254 | 0.009 |
|  | Diet : Testosterone | 2,44 | 3.797 | 0.0301 |
|  | Diet : Testes width | 2,44 | 0.301 | 0.7415 |
|  | Diet : Cortisol : Testes width | 2,44 | 4.834 | 0.0126 |
|  | Diet : Testosterone : Testes width | 2,44 | 3.820 | 0.0295 |

All models initially included ‘diet‘ (control, PUFA, SFA), ‘sex’ (male, female), ‘age’ (second order polynomial), ‘cortisol’ (linear; except for hormone measurements), and ‘litter size’ (linear), as well as up to three-way interactions with diet and sex as fixed effect predictors; ‘age’, ‘individual ID’, and ‘mother ID’ were included as random effects to allow individual growth rates with age and to correct for repeated measurements and relatedness. Non-stated predictors (main effects and interactions) for the respective response variable were removed during model simplification to exclude non-relevant predictors based on the Akaike information criterion.
